# Pharmacokinetic Model-Based Control across the Blood–Brain Barrier for Circadian Entrainment

**DOI:** 10.3390/ijms241914830

**Published:** 2023-10-02

**Authors:** Síofra Ó. Murdoch, Eleonora M. Aiello, Francis J. Doyle

**Affiliations:** 1 Harvard John A. Paulson School of Engineering and Applied Sciences, Harvard University, Boston, MA 02134, USA; sioframurdoch@college.harvard.edu (S.Ó.M.); emaiello@seas.harvard.edu (E.M.A.); 2 Sansum Diabetes Research Institute, Santa Barbara, CA 93105, USA

**Keywords:** circadian rhythm, model predictive control, pharmacockinetics, blood–brain barrier, circadian entrainment

## Abstract

The ability to shift circadian phase in vivo has the potential to offer substantial health benefits. However, the blood–brain barrier prevents the absorption of the majority of large and many small molecules, posing a challenge to neurological pharmaceutical development. Motivated by the presence of the circadian molecule KL001, which is capable of causing phase shifts in a circadian oscillator, we investigated the pharmacokinetics of different neurological pharmaceuticals on the dynamics of circadian phase. Specifically, we developed and validated five different transport models that describe drug concentration profiles of a circadian pharmaceutical at the brain level under oral administration and designed a nonlinear model predictive control (MPC)-based framework for phase resetting. Performance of the novel control algorithm based on the identified pharmacokinetic models was demonstrated through simulations of real-world misalignment scenarios due to jet lag. The time to achieve a complete phase reset for 11-h phase delay ranged between 48 and 72 h, while a 5-h phase advance was compensated in 30 to 60 h. This approach provides mechanistic insight into the underlying structure of the circadian oscillatory system and thus leads to a better understanding of the feasibility of therapeutic manipulations of the system.

## 1. Introduction

Coordinated by the suprachiasmatic nucleus, circadian rhythms are vital, endogenous, approximately 24-h oscillations that synchronize a complex suite of physiological processes, from molecular interactions and expressions to phenotypic and behavioral responses [1,2,3]. To maintain daily periodicity, environmental time cues, including daily light-dark and temperature cycles, serve as entrainers or *zeitgebers* of the period and phase of the endogenous molecular clock and their downstream oscillatory processes [3].

Circadian rhythms play a critical role in controlling core physiological functions such as energy metabolism, fluid balance, inflammation, cellular turnover, and cognition/neurological responses [4]. As a result, any disruption of the rhythm has consequences on human health [5]. Although acute perturbations, such as those caused by light pulses or jet lag, usually decay naturally over seemingly fast time scales spanning a few days [6,7], several studies have identified the disruption of circadian rhythms as a risk-factor in a variety of detrimental health conditions, such as metabolic syndrome, cancer, inflammation, schizophrenia, and cardiovascular disease [8,9,10,11,12]. Due to the short- and long-term health complications associated with circadian misalignment, the problem of driving—or re-entraining—a misaligned circadian oscillator to a reference phase has received a lot of attention [13]. This entrainment problem is typically expressed as a control problem of a nonlinear oscillatory system where circadian dynamics are represented by a limit cycle oscillator that is modeled as a nonlinear dynamical system. In this context, a feedback-loop-based control framework is preferred to open-loop control as it enables more effective regulation in the presence of model uncertainties. Several groups have investigated light-based optimal control or model predictive control (MPC) approaches to manipulate the circadian clock [14,15,16,17]. Such approaches that use light as the control input, however, necessitate several days to complete a phase resetting [13] and often require impractically tight light exposure and avoidance schedules for the subject [18]. As a result, the development of more rapid and more practical mechanisms for the manipulation of these cycles is currently an active research question. Recently, small-molecule pharmaceuticals that target circadian genes have been considered as potential control inputs. Pharmaceuticals offer a number of advantages over light-based control, including rapid clock resetting [19] and increased ease of use. Specifically, by screening a library of ∼60,000 small molecules for their effects on circadian phase, Hirota et al. identified the small molecule KL001 as a potent modulator of circadian rhythms in mammals [20]. According to the Hirota model, the carbazole-containing compound KL001 acts by decreasing the degradation rate of nuclear cryptochrome [20]. As such, the small-molecule pharmaceutical KL001 offers substantial potential for therapeutic action on the circadian phase. To quantify phase changes following perturbation, a phase response curve (PRC) is widely used [21]. These curves map the magnitude and direction of phase shifts resulting from an input. Under the assumption that an input to the clock can be considered to be mediated by temporary changes in one or more parameter values, the input can be represented as a time-dependent modification of parameters, and therefore, the phase response of the circadian clock to therapeutic intervention can be computed using the infinitesimal parametric phase response curve (ipPRC) [22]. Subsequently, the dosing of KL001 can be formulated as a control problem, and recent work has explored the small molecule control of the circadian clock with KL001 as input using the Hirota model [20,23,24].

In [24], the authors designed an ipPRC-based nonlinear MPC algorithm, capable of manipulating mammalian clocks using small-molecule pharmaceuticals. The MPC problem was formulated by reducing a well-established limit cycle model of the circadian clock [25] to a single-order phase reduced model. The work proposed in [24] is limited, however, by the pharmacokinetic representation of the circadian pharmaceutical as a square wave acting directly at the neuron level. This non-physiological approximation does not account for the inherently imperfect and time-dependent process of drug transfer through the body, i.e., from the administration site to the brain. To investigate realistic scenarios, we propose to incorporate and compare different pharmacokinetic (PK) profiles in the aforementioned control framework such that the controlled variable corresponds to the dosage of the pharmaceutical at the site of the administration. We selected three potential PK dynamics and analyzed their impact on the entrainment problem: simple passive diffusion, carrier-mediated transport, and polymeric nanoparticle transcytosis. For the considered pharmacokinetic models, we assumed a normal-release oral administration route given that circadian pharmaceuticals are targeted to be effective, inexpensive, and easily self-administrable. This paper aims to make the following contributions:

- To develop two passive and three active transport models that describe drug concentration profiles of a circadian pharmaceutical at the brain level under oral administration;
- To design an MPC-based control framework based on each PK profile for phase resetting.

The parameters of the proposed PK models are obtained from the literature [26,27,28,29,30] or, when not available, estimated by an extended Kalman filter (EKF) and validated by leveraging the clinical data reported in previous works [31,32,33,34,35]. The performance of the novel control algorithm is demonstrated through simulations of real-world misalignment scenarios due to jet lag, wherein the required time to achieve a complete reset under each physiologically plausible PK input model is compared against the time achieved in the case of an approximation of the PK dynamics by a square wave [24]. To ensure real impact of the case studies, simulations are carried out by considering the full first-principle oscillator model from [20], augmented with the proposed PK model, as a replacement for the real system.

## 2. Material and Methods

In this section, we define the structure of pharmacokinetic models, the parameter identification approach, the circadian oscillator model, and the MPC formulation.

### 2.1. Pharmacokinetic Models

**Figure 1:**
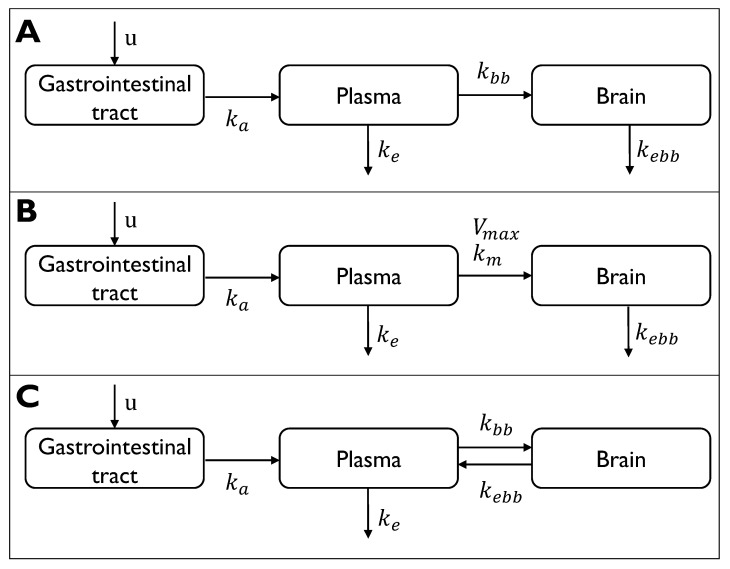
Graphical representation of the three pharmacokinetic models in case of oral input *u*. (**A**) Simple passive diffusion: three-compartment model with linear distribution from gastrointestinal compartment to the brain compartment with intercompartmental rate constants, $k_{a}$ and $k_{bb}$, and with first-order elimination from the plasma compartment and the brain compartment, with elimination rate constants, $k_{e}$ and $k_{ebb}$, respectively. (**B**) Carrier-mediated transport: three-compartment model with linear distribution from gastrointestinal compartment to plasma at constant rate $k_{a}$ and saturable transport from plasma compartment to the brain compartment with $V_{max}$ the maximal elimination rate, and $K_{m}$ is the plasma-concentration at half of the $V_{max}$. First-order elimination processes from the plasma compartment and the brain compartment are included with elimination rate constants, $k_{e}$ and $k_{ebb}$, respectively. (**C**) Polymeric nanoparticle transcytosis: three-compartment model with linear distribution from gastrointestinal compartment to plasma at constant rate $k_{a}$ and reversible kinetics between plasma and the brain compartments with a forward rate constant $k_{bb}$ and reverse rate constant $k_{ebb}$. First-order elimination kinetics from the plasma compartment is included with elimination rate constants, $k_{e}$.

Under the assumption of oral administration, a 3-compartment model was considered to describe the PK dynamics of the drug from the gastrointestinal (GI) system to the brain. The ingested drug (*u*) is defined as an impulse signal whose amplitude corresponds to the effective dosage, which is calculated as the actual dosage (mg) multiplied by the bioavailability of the drug. The drug appears in the first compartment ($x_{1}$), which represents the GI tract. It is enough to assume a single compartment for the GI system as the only value of interest is the drug absorption and elimination process and not how the drug concentration evolves within the compartment. From the GI compartment, the drug is partially absorbed into the plasma compartment ($x_{2}$) and then eliminated with linear elimination. Three different descriptions of the distribution process from the plasma compartment to the brain compartment ($x_{3}$) were developed:

- Simple passive diffusion: linear distribution from the plasma compartment to the brain compartment (Figure 1A);
- Carrier-mediated transport: saturable transport from the plasma compartment to the brain compartment (Figure 1B);
- Polymeric nanoparticle transcytosis: reversible kinetics between the plasma and the brain compartments (Figure 1C).

#### 2.1.1. Passive Diffusion

As numerous studies have demonstrated the regulatory impact of exogenous melatonin on circadian phase timing, it is considered to be a capable chronobiotic for phase shifting mammalian circadian systems [36,37,38,39,40,41]. By virtue of its amphiphilic nature, melatonin rapidly distributes throughout the body via simple passive diffusion [42,43]. Following oral administration, exogenous melatonin is absorbed via passive diffusion from the plasma, across the BBB, into the brain. As such, using the passive diffusion PK model, the dynamics of melatonin, from ingestion to the brain, is given by

(1) $$\begin{array}{l} {\dot{x}}_{1}=-k_{a}x_{1}+Fu\\ {\dot{x}}_{2}=k_{a}x_{1}-k_{e}x_{2}-k_{bb}x_{2}\\ {\dot{x}}_{3}=k_{bb}x_{2}-k_{ebb}x_{3}\\ y_{p}=\frac{1}{V_{p}}x_{2}\\ y_{b}=\frac{1}{V_{b}}x_{3} \end{array}$$

where *u* is the ingested drug (melatonin), $x_{1}$ is the drug mass (mg) in the GI compartment, $x_{2}$ is the drug mass (mg) in the plasma compartment, $x_{3}$ is the drug mass (mg) in the brain compartment, and *F* is the bioavailability of the drug. The parameters $k_{a}$ and $k_{e}$ are the plasma absorption and elimination rate constants (min${}^{-1}$), respectively, $k_{bb}$ is the transport coefficient between the plasma and the brain compartment (min${}^{-1}$), and $k_{ebb}$ is the kinetic constant of drug elimination from the blood–brain barrier (BBB) (min${}^{-1}$). The parameters $V_{p}$ and $V_{b}$ represents the volume (mL) of drug distribution in the plasma and in the brain, respectively. The output $y_{p}$ is the drug concentration in the plasma compartment (mg/mL) and the output $y_{b}$ is the drug concentration in the brain compartment (mg/mL).

For a compact notation, let $x={\left[x_{1}\;x_{2}\;x_{3}\right]}^{T}$, $y={\left[y_{p}\;y_{b}\right]}^{T}$, and define the overall system for passive diffusion ($\Sigma_{PD}$) as

(2) $$\Sigma_{PD}=\left\{\begin{array}{c} \dot{x}=f_{PD}\left(x,u\right)\\ y=g(x). \end{array}\right.$$

#### 2.1.2. Carrier-Mediated Transport

The BBB is characterized by epithelial-like tight junctions, scarce pinocytotic activity and lack of fenestrations (i.e., openings) within the brain capillary endothelium that regulate the traffic of substances into and out of the brain parenchyma [44,45,46]. Due to such characteristics, the barrier prevents entry into the brain of most pharmaceuticals from the blood [47].

Small molecules are capable of crossing the BBB in pharmacologically significant amounts via lipid-mediated diffusion if (1) the molecular mass of the drug is less than 400–500 Da, and (2) the drug forms less than 8–10 hydrogen bonds with solvent water [47,48]. For molecules outside of these specifications, carrier-mediated transport is widely recognized to be the most successful drug delivery method to the brain [46,48]. Specifically, carrier-mediated transport mechanism represents a feasible solution in this context given the molecular structure of the KL001 and the requirement of rapid absorption to complete a phase resetting.

To model specific carrier-mediated transport transcytosis across the BBB, key molecular and structural components of the brain must be considered. Indeed, polarized central nervous system (CNS) endothelial cells (ECs) have distinct luminal and basolateral compartments [49]. The most common and widely utilized BBB model depicts the BBB as a monolayer of highly specialized brain microvascular ECs. This system, known as the Transwell apparatus (e.g., Corning, Lowell, MA, USA), is a vertical side-by-side diffusion system across a microporous semipermeable membrane that separates the luminal and the abluminal compartments [50]. Depending on the pore size of the membrane, cell trafficking across the compartments (generally lumen to albumen) can be enabled. As the capillaries are exposed to luminal pulsatile flow generated by a pumping mechanism, the passage between plasma and brain compartments is described by Michaelis–Menten kinetics [51].

Consequently, for carrier-mediated transport systems, the PK model is defined as follows:

(3) $$\begin{array}{l} {\dot{x}}_{1}=-k_{a}x_{1}+Fu\\ {\dot{x}}_{2}=k_{a}x_{1}-\frac{\left(V_{max}\right)x_{2}}{k_{m}+x_{2}}-k_{e}x_{2}\\ {\dot{x}}_{3}=\frac{\left(V_{max}\right)x_{2}}{k_{m}+x_{2}}-k_{ebb}x_{3}\\ y_{p}=\frac{1}{V_{p}}x_{2}\\ y_{b}=\frac{1}{V_{b}}x_{3} \end{array}$$

where *u* is the ingested drug, $x_{1}$ is the drug mass (mg) in the GI compartment, $x_{2}$ is the drug mass (mg) in the plasma compartment, $x_{3}$ is the drug mass (mg) in the brain compartment, and *F* is the bioavailability of the drug. The parameters $k_{a}$ and $k_{e}$ are the plasma absorption and elimination rate constants (min${}^{-1}$), respectively, $k_{m}$ is the Michaelis constant (mg), which is a measure of substrate-binding affinity, $V_{max}$ (min${}^{-1}$) is the maximum rate of elimination achieved by the system, and $k_{ebb}$ is the kinetic constant of drug elimination from the BBB (min${}^{-1}$). The parameters $V_{p}$ and $V_{b}$ represent the volume (mL) of drug distribution in the plasma and in the brain, respectively. The output $y_{p}$ is the drug concentration in the plasma compartment (mg/mL) and the output $y_{b}$ is the drug concentration in the brain compartment (mg/mL).

For a compact notation, let $x={\left[x_{1}\;x_{2}\;x_{3}\right]}^{T}$, $y={\left[y_{p}\;y_{b}\right]}^{T}$, and define the overall system for carrier-mediated transport ($\Sigma_{CMT}$) as

(4) $$\Sigma_{CMT}=\left\{\begin{array}{c} \dot{x}=f_{CMT}\left(x,u\right)\\ y=g(x). \end{array}\right.$$

Practically, the input *u* must be engineered to meet the chemical requirements necessary to access a given transport system. As such, the possibility of access via adsorptive mediated transcytosis associated with polymeric nanoparticle mediated drug delivery was explored. Polymeric nanoparticles are preferred over non-polymeric ones due to their reduced side effects and ability to increase drug solubility and improve biodistribution [35]. Furthermore, of the eight total neuronal carrier-mediated transport systems [30,46], the amine, neutral amino acid, and triiodothyronine (T3) transport systems were selected in this work as potential inputs.

#### 2.1.3. Polymeric Nanoparticle Transcytosis

The use of charged nanoparticles as a transport mechanism is gaining traction in the field of BBB-pharmaceutical engineering. The model was developed using highly biocompatible Triphenylamine-4-vinyl-(P-methoxy-benzene) (TEB)-based nanoparticles as input, since they can be synthesized as small as 20 nm, which makes them a suitable target for small-molecule delivery [35]. The transport of such nanoparticles at the BBB is governed by first-order reversible kinetics that, following application of mass-action laws, result in the following ODEs:

(5) $$\begin{array}{cc} {\dot{x}}_{1}= & -k_{a}x_{1}+Fu\\ {\dot{x}}_{2}= & +k_{a}x_{1}-k_{bb}x_{2}+k_{ebb}x_{3}\\ {\dot{x}}_{3}= & k_{bb}x_{2}-k_{ebb}x_{3}\\ y_{p}= & \frac{1}{V_{p}}x_{2}\\ y_{b}= & \frac{1}{V_{b}}x_{3} \end{array}$$

where *u* is the ingested drug (TEB), $x_{1}$ is the drug mass (mg) in the GI compartment, $x_{2}$ is the drug mass (mg) in the plasma compartment, $x_{3}$ is the drug mass (mg) in the brain compartment, and *F* is the bioavailability of the drug. The parameters $k_{a}$ and $k_{e}$ are the plasma absorption and elimination rate constants (min${}^{-1}$), respectively, the rate constants $k_{bb}$ and $k_{ebb}$ represent the endocytosis and exocytosis of the nanoparticles across the BBB (min${}^{-1}$), respectively. The parameters $V_{p}$ and $V_{b}$ represents the volume (mL) of drug distribution in the plasma and in the brain, respectively. The output $y_{p}$ is the drug concentration in the plasma compartment (mg/mL), and the output $y_{b}$ is the drug concentration in the brain compartment (mg/mL).

For a compact notation, let $x={\left[x_{1}\;x_{2}\;x_{3}\right]}^{T}$, $y={\left[y_{p}\;y_{b}\right]}^{T}$, and define the overall system for polymeric nanoparticle transcytosis ($\Sigma_{PNT}$) as

(6) $$\Sigma_{PNT}=\left\{\begin{array}{c} \dot{x}=f_{PNT}\left(x,u\right)\\ y=g(x). \end{array}\right.$$

### 2.2. Model Parameters Identification

Several PK variables can be measured experimentally, including maximal plasma concentration ($C_{max}$), time to maximal plasma concentration ($T_{max}$), maximal concentration in the cerebrospinal fluid ($C_{max_{b}}$), time to maximal cerebrospinal fluid (CSF) concentration ($T_{max_{b}}$) elimination half-life ($T_{1/2}$), clearance (CL), area-under-the-curve concentrations (AUC), and the bioavailability (*F*). We used available values of these PK variables to identify the parameters *F*, $k_{a}$, $k_{e}$, $k_{bb}$, $k_{ebb}$, $V_{p}$ in Equations (1), (3), and (5) based on well-established principles of pharmacokinetics [52]. While active transport specific Michaelis–Menten kinetic parameters $k_{m}$ and $V_{max}$ were taken from experimental available values [30], no direct measurement of the volume of distribution ($V_{b}$) in the CSF is available. To obtain an estimate of $V_{b}$, an EKF observer that utilizes noisy CSF concentration measurements was developed [53].

#### Extended Kalman Filter

To estimate the unknown parameter, fictitious parameter dynamics were introduced with the assumption that $V_{b}$ is a constant value. The continuous-time models ($\Sigma_{PD}$, $\Sigma_{CMT}$, and $\Sigma_{PNT}$) are discretized via forward Euler integration with a sampling time of 5 min, obtaining the following discrete-time state-space equations [53]:

(7) $${\tilde{\Sigma }}_{d}=\left\{\begin{array}{cc} x(k+1) & ={\tilde{f}}_{d}\left(x\left(k\right),u\left(k\right)\right)+w^{EKF}\left(k\right)\\ y(k) & =g(x\left(k\right),u\left(k\right)+v^{EKF}\left(k\right) \end{array}\right.\;\;\;\;\;\;\;\mathrm{with}\;d=\left\{PD,CMT,PNT\right\}$$

where *x* is the state vector, *u* is the drug dose, *y* is the measured state, ${\tilde{f}}_{d}\left(\cdot \right)$ is the discrete-time counterpart of the continuous-time dynamic equation f(·), and g(·) is the model output transformation equation, and *k* is the discrete-time instant. The parameter $V_{b}$ is included in the model as a non-measurable, fictitious state with the following dynamics:

(8) $$V_{b}\left(k+1\right)=V_{b}\left(k\right)$$

To obtain an estimate of $V_{b}$, the discrete-time model is augmented as follows:

(9) $$x_{a}\left(k+1\right)=\left[\begin{array}{c} x(k+1)\\ V_{b}\left(k+1\right) \end{array}\right]=\left[\begin{array}{c} {\tilde{f}}_{d}\left(x\left(k\right),u\left(k\right)\right)\\ V_{b}\left(k\right) \end{array}\right]=f_{a}\left(x\left(k\right),u\left(k\right)\right)$$

Process and measurement noises, $w{}_{k}^{EKF}$ and $v{}_{k}^{EKF}$, are defined as follows:

(10) $$\begin{array}{cc} w{}_{k}^{EKF} & ∼N(0,Q{}^{EKF})\\ v{}_{k}^{EKF} & ∼N(0,R^{EKF}) \end{array}$$

where $Q^{EKF}$ is the process noise covariance and $R^{EKF}$ is the measurement noise covariance.

The overall model included in the EKF is then:

(11) $$\left\{\begin{array}{cc} x_{a}\left(k+1\right) & =f_{a}\left(x_{a}\left(k\right),u\left(k\right)\right)+w^{EKF}\left(k\right)\\ y_{a}\left(k\right) & =g_{a}(x_{a}\left(k\right),u\left(k\right)+v^{EKF}\left(k\right) \end{array}\right.$$

At each time instant *k*, the state and measurement Jacobian matrices, $\hat{A}\left(k\right)$ and $\hat{C}\left(k\right)$, respectively, are obtained by linearizing the system dynamics and output transformation models about the point (${\hat{x}}_{a}\left(k|k\right),u\left(k\right)$), as follows:

(12) $$\hat{A}\left(k\right)=\frac{df_{a}\left(x_{a},u\right)}{dx}{|}_{{\hat{x}}_{a}\left(k|k\right),u\left(k\right)}\;\mathrm{and}\;\hat{C}\left(k\right)=\frac{dg_{a}\left(x_{a},u\right)}{dx}{|}_{{\hat{x}}_{a}\left(k|k\right),u\left(k\right)}$$

where ${\hat{x}}_{a}\left(k|k\right)$ is the propagated and updated state from the previous time step and u(k) is the most recent input. The EKF prediction step can be carried out as follows:

(13) $$\begin{array}{cc} {\hat{x}}_{a}\left(k+1|k\right) & =f_{a}\left(x_{a}\left(k|k\right),u\left(k\right)\right)\\ P(k+1|k) & =\hat{A}\left(k\right)P\left(k|k\right)\hat{A}{\left(k\right)}^{T}+Q^{EKF} \end{array}$$

where ${\hat{x}}_{a}\left(k+1|k\right)$ represents the propagated state at the current time step and P(k+1|k) is the state covariance matrix. The measurement, or correction, step for the state estimate and covariance is then performed using the following equations:

(14) $$\begin{array}{cc} K(k+1) & =\hat{A}\left(k\right)P\left(k+1|k\right)\hat{C}{\left(k\right)}^{T}{\left[\hat{C}\left(k\right)P\left(k+1|k\right)\hat{C}{\left(k\right)}^{T}+R^{EKF}\right]}^{-1}\\ {\hat{x}}_{a}\left(k+1|k+1\right) & ={\hat{x}}_{a}\left(k+1|k\right)+K\left(k+1\right)\left[y\left(k\right)-g_{a}\left({\hat{x}}_{a}\left(k+1|k\right)k,u\left(k\right)\right)\right]\\ P(k+1|k+1) & =[I-K\left(k+1\right)\hat{C}\left(k\right)P\left(k+1|k\right)] \end{array}$$

where *K* is the optimal EKF gain, which minimizes the residual error and is dependent upon the current estimation through $\hat{A}\left(k\right)$ and $\hat{C}\left(k\right)$.

### 2.3. Circadian Oscillator Model

The dynamics of circadian systems are often formulated as a deterministic limit cycle oscillator [54,55] that is generally modeled as nonlinear dynamic systems where the states represent the protein concentrations, as proposed in the model from St. John et al. [25]. Since this work is concerned with the phase of the oscillation, the model of St. John et al. [25] can be reduced to a single ODE describing the phase dynamics of the concentration model [56]. Under the assumption that every unique point on the limit cycle solution can be mapped to a unique scalar phase ϕ, the phase-reduced dynamics evolve in time according to

(15) $$\dot{\phi }=\omega +f_{\phi }\left(\phi ,v\right)$$

where ω=2π/T is the angular frequency with period *T* set to 24 h, and $f_{\phi }\left(\cdot \right)$ is the nonlinear function that describes the phase response to a generic input (*v*). Specifically, we approximate $f_{\phi }\left(\cdot \right)$ using the ipPRC, which describes how the phase of the oscillator is affected by an infinitesimal change in parameter for an infinitesimal time. The ipPRC can be numerically derived using sensitivity analysis [56]. The phase equation is therefore given by

(16) $$\dot{\phi }=\omega +B\left(\phi \right)\cdot v$$

where ω is the angular velocity of the oscillator and B(ϕ) is the ipPRC. In this case the input of the phase-reduced model *v* coincides with the drug concentration in the brain obtained by the pharmaceutical input ($y_{b}$). It is worth noting that, in this work, we assume that the oscillator phase is only manipulated by control actions and not perturbed by any other exogenous inputs, such as light.

We refer the reader to St. John et al. [25] for the full set of model equations describing the dynamical states and the reaction in the limit cycle oscillator model, to Abel et al. [24] for the derivation of the single ODE describing the phase dynamics of the oscillator, and to Taylor et al. [56] for the numerical derivation of the ipPRC for the oscillator on the limit cycle.

### 2.4. MPC Formulation

MPC involves predicting the future behavior of the controlled system over a finite time horizon and computing an optimal control input that, while ensuring satisfaction of given system constraints, minimizes an a priori defined cost function.

The MPC problem is formulated in a similar fashion to Abel et al. [24]. While Abel et al. [24] assumed input pharmacokinetics to be approximated by a square wave-which is a non-physiological approximation, we incorporate physiological pharmacokinetics in the optimal control problem.

To estimate the phase of the oscillator in the future, we integrate the ipPRC dynamics as follows:

(17) $$\hat{\phi }\left(t_{i}+\ell \tau \right)=\phi \left(t_{i}\right)+\omega \tau +\sum_{k=1}^{\ell }\int_{t_{i}+\left(k-1\right)\tau }^{t_{i}+k\tau }B\left(\phi \right)\cdot y_{b}dt$$

where $\hat{\phi }$ is the phase estimate, $y_{b}\left(u\right)$ represents the pharmacokinetics, and *u* is the control input at the administration dose.

Let the absolute phase distance $h_{\phi }$ be defined as

(18) $$h_{\phi }=min\left|\hat{\chi }\left(t\right),2\pi -\hat{\chi }\left(t\right)\right|$$

where $\hat{\chi }\left(t\right)=\hat{\phi }\left(t\right)-\phi_{r}\left(t\right)\;mod\;2\pi$ is the predicted phase error from a reference oscillator with phase $\phi_{r}$. To avoid overcorrections due to noise, small phase errors are ignored:

(19) $$g_{\phi }=\left\{\begin{array}{cc} 0 & \mathrm{if}\;h_{\phi }<\delta_{\phi }\\ h_{\phi } & \mathrm{otherwise}. \end{array}\right.$$

The finite-horizon control problem is repeatedly solved at each time $t_{i}$, calculating an optimal trajectory $U^{*}$ over a prediction horizon of length $N_{p}$ with sampling time τ:

(20) $$\begin{array}{c} \begin{array}{cc} u^{*}=\underset{U}{arg min}\sum_{\ell =1}^{N_{p}} & w^{\phi }g_{\phi }^{2}\left(t_{i}+\ell \tau \right)+w_{u}u_{\ell }^{2}\\ \; & \mathrm{subject}\;\mathrm{to}\\ \hat{\phi }\left(t_{i}+\ell \tau \right)= & \phi \left(t_{i}\right)+\omega \tau +\sum_{k=1}^{\ell }\int_{t_{i}+\left(k-1\right)\tau }^{t_{i}+k\tau }B\left(\phi \right)\cdot y_{b}dt\\ \hat{\chi }\left(t\right)= & \hat{\phi }\left(t\right)-\phi_{r}\left(t\right)\\ u_{min}\leq & \sum_{l=1}^{N_{p}}u_{\ell }\leq u_{max} \end{array} \end{array}$$

where $\ell =1,\cdots ,N_{p}$ is the step number, $w^{\phi }$ and $w_{u}$ are positive weighting scalars. The receding horizon (RH) principle is then applied so that the actual control action given as output, $u_{MPC}^{*}$, is the first element of the optimal control sequence $U^{*}$ at each time instant $t_{i}$. The system states and ϕ are assumed to be measurable. For further details on the MPC algorithm, the interested reader is referred to Rawlings et al. [53]. The maximum cumulative control input $u_{max}$ is defined to be consistent with both oral therapeutic administration values for each representative substrate in the gastrointestinal compartment and such that the maximum concentration at the brain level does not exceed a 60% decrease in degradation rate specific to KL001 drug action as this parameter is the dose that acts on the phase of the oscillator. To ensure the controller does not dose beyond therapeutically plausible parameters, when the concentration in the gastrointestinal compartment approaches $u_{max}$, the control input is set to zero in the remaining steps of the prediction horizon. For evaluation, the performance metric includes the time to completion for phase resetting.

## 3. Results

In this section, we present the validation results of the proposed pharmacockinetic models and the controller performance. Different in silico and in vivo studies were considered for identification and validation of the proposed pharmacockinetic models.

### 3.1. Pharmacokinetic Models

Among the available clinical studies, we selected trials investigating pharmacokinetics in healthy subjects via oral administration. We identified the parameters for the kinetic models by utilizing the data reported in Harpsøe et al. [26] and Le Bars et al. [27] for the passive diffusion trasport, in Senek et al. [29] for the carrier-mediated transport, and in Khan et al. [35] for polymeric nanoparticle transcytosis. Active-transport-specific Michaelis–Menten kinetic parameters $k_{m}$ and $V_{max}$ in Equation (3) were taken from experimental values reported in Partridge at al. [30]. The value of $V_{b}$ was estimated via an EKF by using the brain concentration measurements from Van de Berg et al. [28]. The EKF measurement noise covariance $R^{EKF}$ and process covariance matrix $Q^{EKF}$ have been set as follows:

$$R^{EKF}=diag\left(100,100\right),\;Q^{EKF}=diag\left(0,0,0,0.0001\right)$$

The parameter $V_{b}$ was initialized based on the recorded ratio between the AUC of the $y_{p}$ profile and the AUC of the $y_{b}$ profile reported in [57].

Table 1 shows the identified parameters for each transport system. In case of polymeric nanoparticle transcytosis, we assumed the values of *F*, $k_{a}$, $k_{e}$, and $V_{p}$ be the same as the values identified for the passive diffusion model, since a KL001 conjugated to TEB nanoparticle molecule has never been investigated in vivo to our knowledge, and the literature around oral TEB administration is vague.

We validated our PK models by comparing the estimated values of relevant pharmacokinetic parameters, such as the area-under-the-curve plasma concentration ($AUC_{y_{p}}$), maximal plasma concentration ($C_{y_{p}}^{max}$), the area-under-the-curve brain concentration ($AUC_{y_{b}}$), and the maximal brain concentration ($C_{y_{b}}^{max}$), with the available values reported in De Muro et al. [31] for the passive diffusion transport; in Jonklass et al., Bockmann et al., and Nyholm et al. [32,33,34] for the carrier-mediated transport trancytosis; and in Khan et al. [35] for polymeric nanoparticle trancytosis. To validate our carrier-mediated transport model, we used the representative substrate for each system: L-dopa for neutral amino acid transport, choline for amine transport, and triiodothyronine for T3 transport. The value of $C_{y_{p}}^{max}$ was obtained from graphic representation of the data, while the value of $AUC_{y_{p}}$ was calculated according to the trapezoidal rule using the actual times of measurements.

For each clinical study used for validation, Table 2 reports the sample size, the considered transport system with the eventual corresponding substrate, the applied dosage, the measured $AUC_{y_{p}}$ and the estimated ${\hat{AUC}}_{y_{p}}$, and the measured $C_{y_{p}}^{max}$ and the estimated ${\hat{C}}_{y_{p}}^{max}$ on a 12-h simulation scenario. As reported in Table 2, the passive diffusion transport model showed an ${\hat{AUC}}_{y_{p}}$ of 2.31 ×10${}^{-4}$ mg/mL/min, which was similar to the value reported in study [31], while a ${\hat{C}}_{y_{p}}^{max}$ higher than the reference value was achieved. It is worth noting that De Muro et al. reported a range of $C_{y_{p}}^{max}$ between 2.2 ×10${}^{-6}$ mg/mL and 5.7 ×10${}^{-6}$ mg/mL across 12 participants and the estimated ${\hat{C}}_{y_{p}}^{max}$ of 4.67 ×10${}^{-6}$ mg/mL was well within the range of all values. In the case of the T3 pharmacokinetics, there was no difference between the values of $AUC_{y_{p}}$ and ${\hat{AUC}}_{y_{p}}$ and the values of $C_{y_{p}}^{max}$ and ${\hat{C}}_{y_{p}}^{max}$, as shown in Table 2. We estimated a time to maximal plasma concentration of 150 min, which is in agreement with the range reported by Jonklaas et al., i.e., between 120 and 240 min across 12 subjects [32]. Similar values to the measured $AUC_{y_{p}}$ and $C_{y_{p}}^{max}$ were obtained for choline, as shown in Table 2. For the neutral amino acid, while the ${\hat{AUC}}_{y_{p}}$ value of 0.12 mg/mL/min was above the experimentally recorded average of 0.08 mg/mL/min, ${\hat{AUC}}_{y_{p}}$ falls within one standard deviation, which is equal to 0.04 mg/mL/min [34]. Finally, since no relevant in vivo experimental data could be found, the polymeric nanoparticle model was validated against the reported values of the parameters ${AUC}_{y_{b}}$ and $C_{y_{b}}^{max}$, which were measured for in vitro experimental data at the brain level and reported by Khan et al. [35]. The polymeric nanoparticle model matched the in vitro parameters well, with the estimated AUC of concentration in the brain compartment (${\hat{AUC}}_{y_{b}}$) of 3.24 ×10${}^{-3}$ mg/mL/min against an ${AUC}_{y_{b}}$ of 3.27 ×10${}^{-3}$ mg/mL/min value from experimentally recorded values. The estimated peak concentration in the brain compartment (${\hat{C}}_{y_{b}}^{max}$) was 8% less than $C_{y_{b}}^{max}$, i.e., 5.97 ×10${}^{-3}$ mg/mL vs. 5.48 ×10${}^{-3}$ mg/mL, respectively.

**Figure 2:**
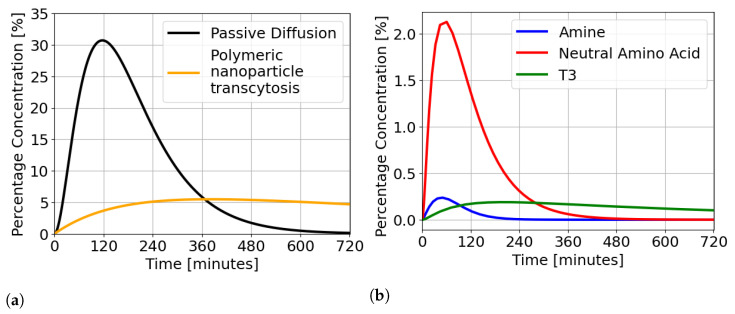
Concentration profiles in the brain compartment obtained with a unit of percentage of the administered dose. (**a**) Concentration profiles in the cases of passive diffusion model (black) and polymeric nanoparticle transcytosis transport (orange). (**b**) Concentration profiles for the three carrier-mediated transport models: amine (blue), neutral amino acid (red), and T3 (green).

As shown in Table 2, the reported therapeutic dosages for the different representative substrates ranged from 0.001 up to 600 mg, and identified values of $V_{b}$ are model-related, as reported in Table 1. To deal with this issue and obtain a fair comparison among the proposed PK models, we compared the estimated concentrations in the brain compartment to a standardized unit of percentage of administered dose (${\hat{y}}_{b}^{\%}$), where the administered doses remained consistent with values indicated in Table 2. For each transport mechanism, the obtained profiles of ${\hat{y}}_{b}^{\%}$ are displayed over a uniform 12-h time interval in Figure 2. Figure 2a shows the curves of ${\hat{y}}_{b}^{\%}$ in the passive diffusion transport and the polymeric nanoparticle transcytosis, while Figure 2b displays the curves of ${\hat{y}}_{b}^{\%}$ for the three carrier-mediated transport models—amine, neutral amino acid, and T3 transport systems. The transport systems in Figure 2a display a far greater percentage of administered dose that reaches the brain as compared to the carrier-mediated transport models (Figure 2b). In the case of passive diffusion, the maximum percentage of the administered dose reaching the brain is 30% against a percentage of 5% for polymeric nanoparticle transcytosis and smaller percentages between 0.23% and 2.12% for the three carrier-mediated transport models. As reported in Table 3, conversely, the observed carrier-mediated transport kinetics are faster, with peak times between 50 and 190 min, which is consistent with the hypothesis that carrier-mediated transport offers a mechanism to speed up passage across the BBB and supports the idea that carrier-mediated transport mechanisms represent a keen target for neural pharmaceuticals that require rapid or frequent dosing [58].

### 3.2. Controller Performance

To assess the efficacy of the three pharmaceutical process models to the control approach, a simulation scenario, involving a 5-h phase advance followed three days after by an 11-h phase delay, was carried out using the full oscillator model from [20,25] as replacement for the real system. For each PK model evaluation, the full oscillator model was first augmented with the PK model under consideration. This choice allowed evaluation of the control performance under model/plant mismatch, which is always the case in the real world application. For comparison purposes, the simulation scenario is consistent with the scenario proposed in Abel et al. [24], which will be referred to hereafter as the “baseline.” For the simulation, we parametrized the controller with a sampling time of τ=2 h, a prediction horizon consisting of $N_{p}=3$, i.e., 6 h, weight on the absolute phase error $w_{\phi }=1$, the lower bound on the cumulative control input $u_{min}=0$, and the threshold $\delta_{\phi }=0.1$. The value of $u_{max}$, which represents the upper bound on the cumulative control input, was selected within feasible physiological ranges to reflect both therapeutically plausible oral administration dosages and the 60% decrease in degradation rate specific to KL001 drug action at the brain level [25]. As a result, the weight associated with control input $w_{u}$ was tuned for each transport system to adjust the aggressiveness of the controller. The values for $u_{max}$ and $w_{u}$ are reported in Table 4. Given the small percentage of the drug concentration reaching the brain in case of carrier-mediated transport, as shown in Figure 2b, a small $w_{u}$ was selected to increase the aggressiveness of the controller.

**Figure 3:**
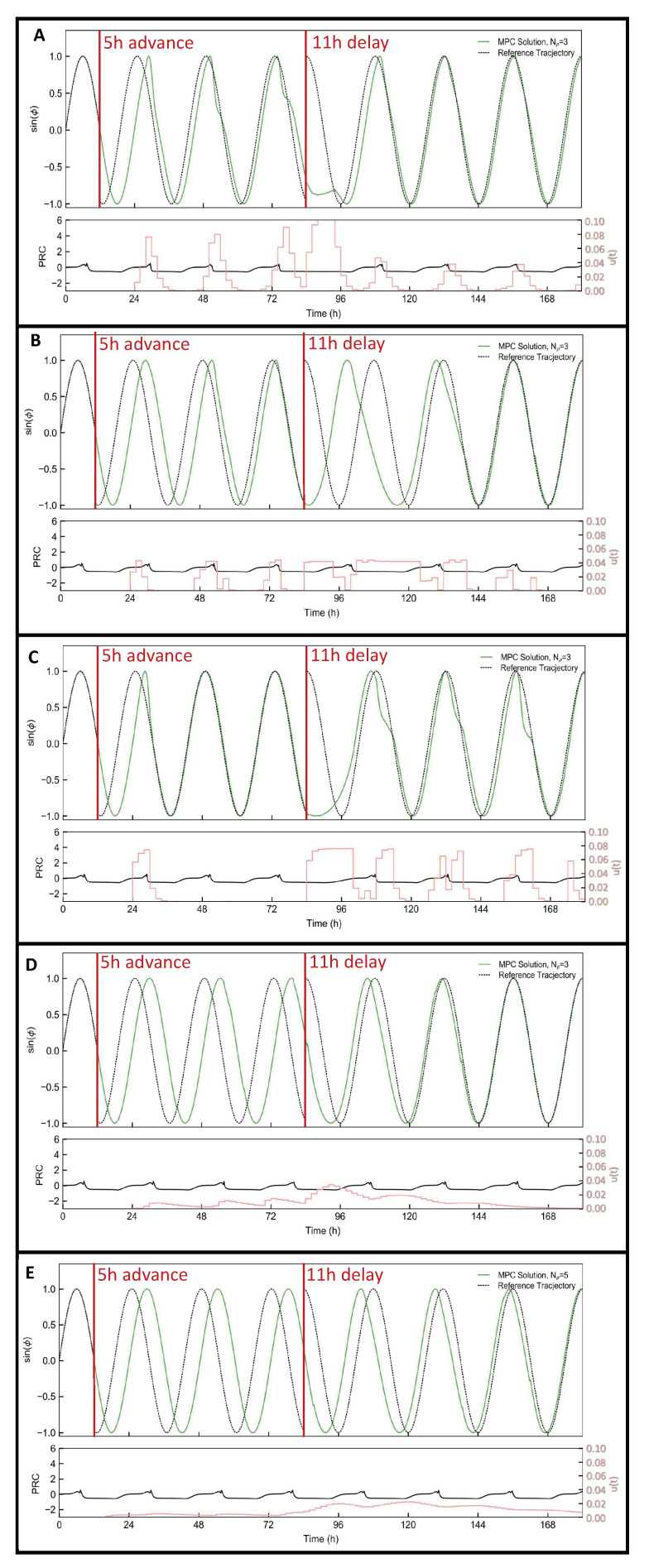
Results of MPC for phase resetting on the simulation scenario, involving a 5-h phase advance followed by 11-h phase delay three days later are reported for (**A**) passive diffusion, (**B**) carrier-mediated transport (Amine), (**C**) carrier-mediated transport (Neutral Amino Acid), (**D**) carrier-mediated transport (T3), and (**E**) polymeric nanoparticle transcytosis. In the top panel, the phase of the oscillator under MPC in green is compared with the reference phase (local time) in black. In the bottom panel, the corresponding ipPRC is reported in black, while the optimal control inputs (*u*) are reported in pink with the axis on the right side.

The time to achieve a complete phase reset under each pharmacokinetic administration model is reported in Table 4 alongside the baseline formulation. It should be noted that achieving phase reset in less than 48 h represents a substantial improvement in the capacity of natural light to re-entrain circadian phase in jet lag scenarios, wherein the approximate rate of realignment is only 1 h of time zone change per day [59]. The controllers designed for the polymeric nanoparticle transcytosis and the T3-carrier-mediated transport models were not able to complete the reset of the phase advance. Additionally, as shown in Figure 3E, the smaller pharmaceutical dosages that reach the brain and the slower pharmacokinetics of nanoparticle adsorptive transcytosis result in reduced capacity to achieve rapid phase reset. Further, slower clearance rates in the T3-carrier-mediated transport administration model resulted in a costly over-eagerness to dose. Without allowing sufficient time between doses to clear drug concentration, the controller dosed continuously, causing a build-up of drug as a result of the delayed kinetics of physiological dosing. This compounding effect resulted in saturating the control action to $u_{max}$ and consequent degradation of the circadian oscillator, as shown in Figure 3D. In the case of passive diffusion, the controller completes the 5-h phase advance and 11-h phase delay in approximately 60 and 66 h, respectively, as reported in Table 4. The passive diffusion model does appear to be insufficient for the target case of circadian phase resetting, which demands rapid kinetics for satisfactory performance. The controller designed for the amine-carrier-mediated transport input model realigned the phase in approximately 50 h, while the best performance was achieved in the case of input governed by neutral-amino-acid-carrier-mediated transport pharamcokinetics. In the latter case, the controller was able to successfully realign the oscillator in 30 and 48 h in the case of phase advance and delay, respectively, requiring only an additional 6 h compared to the baseline MPC problem, as reported in Table 4.

## 4. Discussion

Though the field of engineering BBB transport mechanisms is still in its infancy, a major focus of research surrounds identifying strategies to enhance the delivery of therapeutics across the BBB [49]. Comparative analysis of the phase shifting responses under varied transport mechanism reveals key insights in this direction with focus on the mechanistic processes underlying circadian rhythm regulation and function. Brown et al. [60] have described disparity in period sensitivity between positive and negative feedback loops with the hypothesis that small-molecule drug development should focus on molecules that target the negative feedback loop for rapid circadian phase shifting. MPC resetting results for amine-carrier-mediated transport and neutral-amino-acid-carrier-mediated transport pharmacokinetics provide important support to the hypothesis proposed in [60]. A limitation of the proposed structure of the carrier-mediated transport pharmacokinetics is the assumed absence of hydrostatic or osmotic pressure difference across the barrier [45]. Since the temporal effects of such dynamics are presumed to be minimal, this premise represents a trade-off between an effective system description and the elevated complexity of the actual system under analysis. Still, the model accounts for the range of pertinent transport systems through the modification of active permeability parameters that are specific to particular transporters [51]. The main challenge confronted in the model design, however, was the lack of availability in the literature of cerebrospinal fluid concentration data for any of the representative substrates described by the carrier-mediated transport mechanism. As such, the approximation of these values represents the primary simplification of the system. Another challenge was the absence of specific guidelines for the recommended drug dosages for each of the five proposed PK input mechanisms. As a result, this demanded a conservative approach to control parametrisation to avoid an excessive dosing strategy at the occurrence of the phase shift. Future work may consider introducing a sparsity constraint to induce a safer dosing strategy in terms of toxicity, or dose–response side effect profiles, towards the applicability of the proposed approach in an in vivo study. Future work will also include the analysis of the impact of constant rates on the MPC performance, including additional administration routes, such as transnasal or intravenous.

## 5. Conclusions

In this work, we investigated therapeutic strategies that allow for the rapid re-entrainment of a circadian oscillator to its environment via nonlinear MPC. To evaluate the performance of the controller under realistic conditions, we modeled five different pharmacokinetic behaviors associated with therapeutic administration. This approach allows us to provide mechanistic insight into the underlying structure of the circadian oscillatory system and, thus leads to a better understanding of the feasibility of therapeutic manipulations of the system. Furthermore, as the emerging field of BBB research intently focuses on methods to enhance the delivery of therapeutics, the development of the proposed models plays a key role in understanding future BBB transport possibilities and limitations.

## Figures and Tables

**Table 1:** Parameters of the pharmacokinetic models. The parameters for the kinetic models were identified by utilizing the data reported in Harpsøe et al. [26], Le Bars et al. [27] and van der Berg et al. [28] for the passive diffusion trasport, in Senek et al. [29] and Partridge et al. [30] for the carrier-mediated transport transcytosis, and in Khan et al. [35] for polymeric nanoparticles. The values of $V_{b}$ were estimated via extended Kalman filter.

Transport System	*F* [%]	$k_{a}$ [min${}^{-1}$]	$k_{e}$ [min${}^{-1}$]	$k_{\mathrm{bb}}$ [min${}^{-1}$]	$k_{\mathrm{ebb}}$ [min${}^{-1}$]	$V_{p}$ [mL]	$V_{b}$ [mL]	$V_{\max}$ [mg/min]	$k_{m}$ [mg/mL]
Passive diffusion [26,27,28]	0.14	0.11	0.11	4.53 ×10${}^{-2}$	2.82 ×10${}^{-2}$	1.80 ×10${}^{5}$	3.30 ×10${}^{5}$	-	-
Carrier-mediated transport (Amine) [29,30]	0.80	2.90 ×10${}^{-2}$	0.10	-	3.38 ×10${}^{-2}$	3.00 ×10${}^{4}$	0.90 ×10${}^{4}$	10.00	0.44
Carrier-mediated transport (Neutral Amino Acid) [29,30]	0.30	1.40 ×10${}^{-2}$	1.30 ×10${}^{-2}$	-	2.82 ×10${}^{-2}$	1.00 ×10${}^{4}$	0.30 ×10${}^{4}$	30.00	0.12
Carrier-mediated transport (T3) [29,30]	0.75	4.00 ×10${}^{-3}$	0.02	-	1.30 ×10${}^{-2}$	1.50 ×10${}^{4}$	3.00 ×10${}^{4}$	0.10	1.00 ×10${}^{-3}$
Polymeric nanoparticle transcytosis [35]	0.14	0.11	0.11	6.10 ×10${}^{-3}$	8.30 ×10${}^{-4}$	1.80 ×10${}^{5}$	3.30 ×10${}^{5}$	-	-

**Table 2:** Baseline characteristics of the clinical studies used for validation purposes. For each study, the sample size, the considered transport system with the eventual corresponding substrate, the applied dosage, the measured and the estimated area under the plasma concentration–time curves from zero to the next 12 h, ${\hat{AUC}}_{y_{p}}$, and ${AUC}_{y_{p}}$ and the reported and the estimated maximum observed plasma concentration $C_{y_{p}}^{max}$ and ${\hat{C}}_{y_{p}}^{max}$ are reported.

Reference	Sample Size	Trasport System (Substrate)	Dose [mg]	${\mathrm{AUC}}_{y_{p}}$ [mg/mL/min]	${\hat{\mathrm{AUC}}}_{y_{p}}$ [mg/mL/min]	$C_{y_{p}}^{\max}$ [mg/mL]	${\hat{C}}_{y_{p}}^{\max}$ [mg/mL]
De Muro et al. [31]	12	Passive diffusion	2.00	2.37×10${}^{-4}$	2.31 ×10${}^{-4}$	3.40 ×10${}^{-6}$	4.67 ×10${}^{-4}$
Jonklass et al. [32]	12	T3 (Triiodothyronine)	0.05	1.12 ×10${}^{-3}$	1.94 ×10${}^{-3}$	3.46 ×10${}^{-6}$	3.81 ×10${}^{-6}$
Bockmann et al. [33]	6	Amine (Choline)	550.00	0.40	0.30	1.04 ×10${}^{-3}$	2.30 ×10${}^{-3}$
Nyholm et al. [34]	19	Neutral amino acid (L-Dopa)	100.00	8.48 ×10${}^{-2}$	0.12	9.46 ×10${}^{-4}$	1.13 ×10${}^{-3}$

**Table 3:** Comparison of the maximum percentage of the administered dose reaching the brain and peak time in the brain following oral administration for passive diffusion, carrier-mediated transport (amine, neutral amino acid, and T3) and polymeric nanoparticle transcytosis.

Transport System	% Administered Dose	$T_{\max}$ [min]
Passive diffusion	30.70	117
Carrier-mediated transport (Amine)	0.23	50
Carrier-mediated transport (Neutral Amino Acid)	2.12	61
Carrier-mediated transport (T3)	0.18	193
Polymeric nanoparticle transcytosis	5.48	380

**Table 4:** Tuning parameters of the model predictive control (MPC) problem, including the upper bound for the control sequence $u_{max}$, and the weight on input $w_{u}$, together with the times to achieve a complete reset in the simulation scenario, involving a 5-h phase advance followed by an 11-h phase delay, are reported for each transport system. N/A is reported if the reset has never been achieved.

Transport System	$u_{\max}$[mg]	$w_{u}$	Time Required for 5-h Phase Advance [h]	Time Required for 11-h Phase Delay [h]
Passive diffusion	2.00	1.00	60	66
Carrier-mediated transport (Amine)	16.50	1.00 ×10${}^{-3}$	50	54
Carrier-mediated transport (Neutral Amino Acid)	3.00	0.10	30	48
Carrier-mediated transport (T3)	4.30	0.10	N/A	72
Polymeric nanoparticle transcytosis	0.06	1.00	N/A	84
Baseline [24]	0.06	1.00	30	36

## Data Availability

Not applicable.
